# Does butylphthalide affect on hemodynamics in patients with watershed stroke? A protocol of systematic review and meta-analysis

**DOI:** 10.1097/MD.0000000000020151

**Published:** 2020-05-15

**Authors:** Li-na Jia, Ya-juan Zhang, Rong Ma, You Song

**Affiliations:** a Department of Neurology, The Central Hospital of Jia Mu Si City, Jiamusi; b Department of Neurology, First Affiliated Hospital of Jiamusi University, Jiamusi, China.

**Keywords:** butylphthalide, effect, hemodynamics, watershed stroke

## Abstract

**Background:**

This study will specifically investigate the effect of butylphthalide on hemodynamics in patients with watershed stroke (WS).

**Methods:**

We will search the following databases from their inceptions to the March 1, 2020: Cochrane Library, MEDLINE, EMBASE, PsycINFO, Web of Science, Cumulative Index to Nursing and Allied Health Literature, and China National Knowledge Infrastructure. All relevant randomized controlled trials on exploring the effect of butylphthalide on hemodynamics in patients with WS will be considered for inclusion. No language limitation will be imposed to this study. All study quality will be checked using Cochrane risk of bias tool. RevMan 5.3 software will be utilized for data analysis.

**Results:**

This study will summarize the latest evidence to investigate the effect of butylphthalide on hemodynamics in patients with WS.

**Conclusion:**

Findings from this study will provide theoretical basis of butylphthalide on hemodynamics in patients with WS for clinician and future research.

**Dissemination and ethics:**

This study is carried out based on the published data, thus, no ethical approval is required. We will submit this study to a peer-reviewed journal for publication.

**Systematic review registration:**

INPLASY 202030006.

## 1 Introduction

Stroke is very common, harmful, and dangerous long-term neurological disability and mortality disorder around the world.^[1,2,3,4]^ It includes ischemic stroke and hemorrhagic stroke.^[5,6,7]^ Of those, watershed stroke (WS) accounts for 10% to 64% of all ischemic stroke.^[8,9,10]^ It is also relevant with a high risk of cerebrovascular disease and its recurrence.^[11,12]^ It is characterized as localized to the vulnerable border zones, which supply a reduction of blood flow and oxygen to the brain.^[13,14,15,16]^ Therefore, hemodynamics accurately shows the severe conditions of WS.

Previous studies have reported that butylphthalide can effectively impact hemodynamics in patients with WS.^[17,18,19,20,21,22]^ However, there are still not consistent results among those studies. In addition, no systematic review has investigated the effect of butylphthalide on hemodynamics in patients with WS. Thus, this study will systematically explore the effect of butylphthalide on hemodynamics in patients with WS.

## 2 Methods

### 2.1 Study registration

We have registered this study on International Platform of Registered Systematic Review and Meta-Analysis Protocols (INPLASY 202030006) (https://inplasy.com/wp-content/uploads/2020/03/INPLASY-Protocol-06.pdf). It has been reported follows the guidelines of Preferred Reporting Items for Systematic Review and Meta-Analysis Protocols Statement.^[23]^

### 2.2 Eligibility criteria

#### 2.2.1 Types of trials

This study will include randomized controlled trials (RCTs) that focus on exploring the effect of butylphthalide on hemodynamics in patients with WS.

#### 2.2.2 Types of patients

This study will include patients who were diagnosed as WS regardless their race, gender, and age.

#### 2.2.3 Types of interventions

In the intervention group, all eligible WS patients received butylphthalide on hemodynamics.

In the control group, all included WS participants underwent any other management, but not butylphthalide.

#### 2.2.4 Types of outcome measurements

The primary outcomes are hemodynamic indices, as measured by mean velocity of blood flow, flow velocity, mean blood flow, cardiac output, and blood pressure.

The secondary outcomes include cerebral blood flow, cerebral blood volume, mean transit time, quality of life, and any adverse events.

### 2.3 Information sources and search strategy

Cochrane Library, MEDLINE, EMBASE, PsycINFO, Web of Science, Cumulative Index to Nursing and Allied Health Literature, and China National Knowledge Infrastructure will be searched from inception to the March 1, 2020. We will not impose any language and publication status limitations. All potential RCTs on exploring the effect of butylphthalide on hemodynamics in patients with WS will be included in this study. The Cochrane Library search strategy is shown in Table 1. We will also adapt similar search strategy to the other electronic databases. In addition, we will perform searches in conference abstracts and reference lists of eligible studies.

**Table 1:**
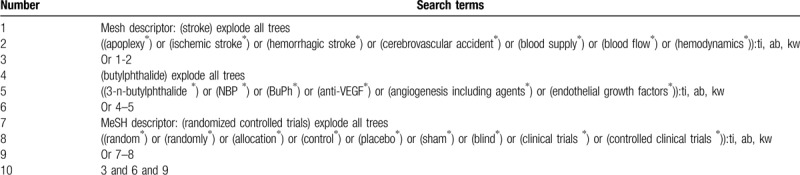
Search strategy for Cochrane Library.

### 2.4 Study selection

Two investigators will independently check the titles and abstracts of all searched records, and all non-associated records will be removed. Then, we will check the full papers of remaining records and will further judge if they finally meet all inclusion criteria. Any different opinions between two investigators will be solved by a third experienced investigator through consultation. Any unqualified literatures will be noted with specific reasons for exclusion. The process of study selection will be exerted in a flowchart in this study.

### 2.5 Data extraction and management

Two investigators will independently extract data from included studies based on the predefined standardized form for data collection. Any discrepancies between two investigators will be solved by a third expert investigator through discussion. Extracted information includes study basic information (including title, first author, et al), patient characteristics (including age, race, gender, et al), study setting, sample size, study methods (including details of randomization, blind, et al), intervention and comparators (including types, dosage, mode of delivery, et al), outcomes (any outcome measurements, safety, et al), and funding information.

### 2.6 Missing data management

If we identify any missing data or unclear information during the period of data extraction, we will contact original authors to inquire those data. If we cannot receive those data, we will analyze the available data only.

### 2.7 Risk of bias assessment

Study methodological quality will be measured using Cochrane risk of bias tool through risk bias of selection, performance, detection, attrition, reporting, and others. Each item is further assessed as high, unclear or low risk of bias. Two independent reviewers will assess the methodological study quality for all included studies. A third experienced investigator will help to solve all disagreements between two investigators.

### 2.8 Statistical analysis

In this study, we will use RevMan 5.3 software for statistical analysis. We will calculate all binary data using risk ratio and 95% confidence intervals (CIs), and all continuity data using mean difference, or standardized mean difference and 95% CIs. Heterogeneity among studies will be checked by *I*^2^ test. The value of *I*^2^ ≤ 50% exerts minor heterogeneity, while the value of *I*^2^ > 50% shows obvious heterogeneity. If *I*^*2*^ ≤ 50%, we will use a fixed-effects model, and will conduct meta-analysis if sufficient data on the same outcomes are collected. If *I*^*2*^ > 50%, we will utilize a random-effects model, and will carry out subgroup analysis or meta-regression test to check any potentials factors for obvious heterogeneity.

### 2.9 Additional analysis

#### 2.9.1 Subgroup analysis

Data permitting, we will perform subgroup analysis considering different study information, patient characteristics, interventions, controls, and outcome measurements.

#### 2.9.2 Sensitivity analysis

If sufficient available data is extracted, we will plan to conduct sensitivity analysis to check the stability for the outcome results by excluding low methodological quality studies.

#### 2.9.3 Reporting bias

Publication bias will be identified via Funnel plot and Egger's regression test if sufficient studies are included.^[24,25]^

## 3 Discussion

This study will explore whether butylphthalide effectively impact the hemodynamics in the participant with WS. This study will provide a concise resource of current evidence of butylphthalide on the hemodynamics in patients with WS. Its findings may help to provide reference for clinicians, policy makers, stakeholders, and researchers. It may also help to identify if there are any research gaps and opportunities for the further studies.

## Author contributions

**Conceptualization:** You Song, Li-na Jia, Ya-juan Zhang.

**Data curation:** Li-na Jia, Ya-juan Zhang, Rong Ma.

**Formal analysis:** Li-na Jia, Ya-juan Zhang, Rong Ma.

**Funding acquisition:** You Song.

**Investigation:** You Song.

**Methodology:** Rong Ma.

**Project administration:** You Song.

**Resources:** Li-na Jia, Ya-juan Zhang, Rong Ma.

**Software:** You Song, Li-na Jia, Ya-juan Zhang, Rong Ma.

**Supervision:** You Song, Rong Ma.

**Validation:** You Song, Li-na Jia, Ya-juan Zhang, Rong Ma.

**Visualization:** You Song, Li-na Jia, Rong Ma.

**Writing – original draft:** You Song, Li-na Jia, Ya-juan Zhang, Rong Ma.

**Writing – review & editing:** You Song, Li-na Jia, Ya-juan Zhang, Rong Ma.
